# Implementation of the Su–Schrieffer–Heeger Model in the Self-Assembly Si–In Atomic Chains on the Si(553)–Au Surface

**DOI:** 10.1021/acsnano.4c00225

**Published:** 2024-05-07

**Authors:** Mieczysław Jałochowski, Mariusz Krawiec, Tomasz Kwapiński

**Affiliations:** Institute of Physics, Maria Curie-Sklodowska University, Lublin 20-031, Poland

**Keywords:** atomic chains, stepped surface, STM spectroscopy, SSH chain, topology, density od states

## Abstract

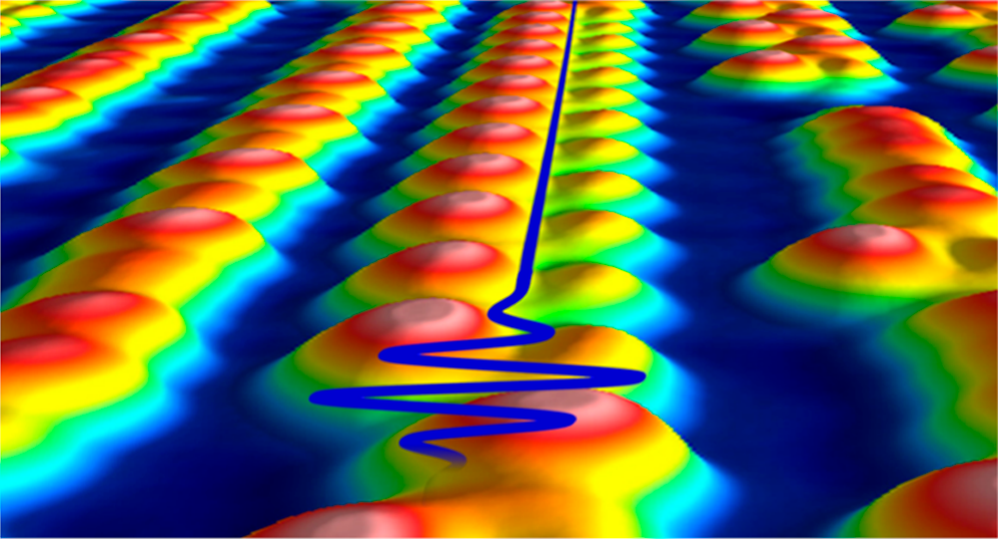

Indium-decorated Si atomic chains on a stepped Si(553)–Au substrate are proposed as an extended Su–Schrieffer–Heeger (SSH) model, revealing topological end states. An appropriate amount of In atoms on the Si(553)–Au surface induce the self-assembly formation of trimer SSH chains, where the chain unit cell comprises one In atom and two Si atoms, confirmed by scanning tunneling microscopy images and density functional calculations. The electronic structure of the system, examined through scanning tunneling spectroscopy, manifests three electron bands within the Si–In chain, accompanied by additional midgap topological states exclusively appearing at the chain’s end atoms. To elucidate the emergence of these topological states, a tight-binding model for a finite-length-extended SSH chain is proposed. Analysis of the energy spectra, density of states functions, and eigenfunctions demonstrates the topological nature of these self-assembled atomic chains.

## Introduction

Atomic chains serve as ultrathin electrical conductors with wide applications.^1−4^ In these one-dimensional (1D) systems, various quantum phenomena have been observed, including spin-charge separation,^5^ Friedel oscillations,^6^ charge-density waves,^7,8^ fractional charges,^9^ and transient crystals,^10^ among others. Recently, topological materials have garnered significant interest due to their unique characteristics—they act as insulators in bulk but harbor midgap boundary states. In 1D systems, these boundaries exist solely at both ends, akin to Majorana topological states,^11,12^ making such materials particularly fascinating.

One of the simplest realizations of a topological 1D system is the Su–Schrieffer–Heeger (SSH) model,^13−17^ initially proposed to describe modified polyacetylene chains in chemistry. Essentially, the SSH model represents a dimerized chain with alternating couplings within and between dimers. This system possesses time-reversal particle-hole symmetry and supports two distinct topological phases. Nontrivial topology is also observed in extended SSH models featuring various geometries, such as long-range chains incorporating next-nearest-neighbor hoppings or by altering the site period of the unit cell.^14,18−24^ Moreover, the standard SSH model extends to a double-chain structure, resembling a cross-linked two-leg ladder model (Creutz ladder) or a modified Harper model.^25−28^ Topological states can also emerge in two-dimensional (2D) ribbon geometries or disrupted SSH chains.^29−33^ Intriguingly, ladder-like systems can unveil topological Majorana states^34^ as well. Additionally, nontrivial phases of matter are observed in driven SSH chains, sometimes termed Floquet topological insulators.^35,36^ Numerous potential experimental applications of the SSH chain are observed in the domains of quantum optics and momentum lattices, wherein dynamic effects can generate nontrivial 1D structures featuring midgap topological states.^18,37,38^ While this approach demands sub-Kelvin temperatures, it enables the creation of Creutz ladder systems or extended SSH structures using ultracold Fermionic atoms.^18,25^ In these structures, the estimation of topological properties is conducted through quench dynamics. The successful implementation of the atomic model of SSH in the form of a kind of “negative” chain dimer was made from vacancy defects in the chlorine superstructure c(2 × 2) on Cu(100).^39^ The network of coupled states was built from states below the band edge of the chlorine layer. A similar system has been used to study coupled dimer chains with domain wall states.^40^

Presently, atomic manipulation methods have become instrumental in fabricating SSH 1D structures. For instance, a series of quantum dots, each composed of six atoms, was considered as a topological system.^41^ Such periodical chains can be easily described within the tight binding (TB) model, which can be straightforwardly mapped onto a modified SSH geometry. However, it is noteworthy that in this study, the relative distance between the dots was quite substantial, approximately 5 nm, seemingly too large for a direct overlap of neighboring dots’ wave functions. Similarly, Si chains on the Si(553) surface can form trimer 1D structures exhibiting solitons and fractional charges.^8,9^ However, the hopping integrals along these chains are remarkably similar, leading to relatively small energy gaps in the energy spectra, thus inhibiting the emergence of topological end states in this scenario.

It is also important to note the observation of 1D charge density waves with chiral solitons, akin to topological materials, in a coupled double Peierls chain of In atoms on the Si surface.^42^ This observation highlights the diverse behaviors and potential topological properties exhibited by different atomic arrangements and structures. As far as our knowledge extends, the practical implementation of self-assembled chains in the nontrivial geometry of SSH was not reported in the literature. Although the existence of topological end states is anticipated in finite-length structures, these nontrivial states have not been observed in atomic systems such as pure Si or In chains.^8,9,42,43^ One promising candidate for such an implementation could be the Si atomic chain forming stepped edges on vicinal surfaces of Si(111), stabilized with Au atoms.^44,45^ Our earlier calculations indicated that topological SSH states can persist in the presence of various substrate electrodes.^46^ Furthermore, these states can also be observed in atomic chains arranged on stepped surfaces. Therefore, in this study, we investigate the Si(553)–Au surface hosting nontrivial Si chains^8,9^ and decorate this structure by incorporating a small amount of In atoms to obtain finite-length Si–In atomic chains. Note that the electronic structure of Si(335)–Au surface is weakly hybridized with the In atoms,^47^ thus the Si–In chains stand for 1D structures in the modified SSH geometry. The presence of In atoms arranged along the Si chains might either entirely disrupt the topological phase or enhance the topological states by increasing the energy gaps in the system. In this letter, we address this issue through scanning tunneling spectroscopy (STS) investigations of Si–In chains, and our findings are corroborated by theoretical density functional theory (DFT) and TB studies.

Significant research has been dedicated to examining the atomic and electronic structures of the Si(553)–Au surface.^8,48−57^ An earlier theoretical model^51^ with periodicity ×2 along the step edge suggested that three Si orbitals of each edge atom form covalent bonds with neighboring Si atoms, while the fourth orbitals create a chain with ×1 periodicity. Additional DFT calculations and scanning tunneling microscopy (STM) measurements have indicated that the periodicity of the Si chain, characterized by × 3 periodicity in the ground state, can shift to ×2 periodicity upon electron injection via STM tip, which is also associated with temperature changes in the sample.^52^ Further refinement of the model was proposed in ref (43), wherein a transition of the Si(553)–Au surface from 2D order to 1D behavior was observed. This transition was attributed to the creation of phase solitons and antisolitons at a characteristic temperature of around 100 K. The surface also exhibits a ground state with triple degeneracy in Si chains, representing an insulator with Z_3_ topology.^8^ Moreover, modification of this surface by external atoms is a subject of study in the literature; for instance, after the deposition of In atoms, a hybrid Si–In chain can form on the surface steps.^47,58^ Consequently, the Si(553) surface has emerged as a promising platform for synthesizing self-assembled topological chains.

## Results and Discussion

This study presents experimental evidence of topological SSH states in self-assembled regular chains on the terraces of the Si(553)–Au surface. These chains consist of Si and In atoms, representing an extended SSH chain (SSH3 geometry with three atomic sites in each unit cell). The relatively low coverage of In atoms allows for the fabrication of finite-length Si–In SSH chains, revealing topologically protected end states. Hence, this combined experimental and theoretical work reveals the nontrivial topological nature in real-atom systems fabricated via controllable self-assembly methods.

### Topography and Electronic Structure of Si–In Chains on Si(553)–Au

**Figure 1:**
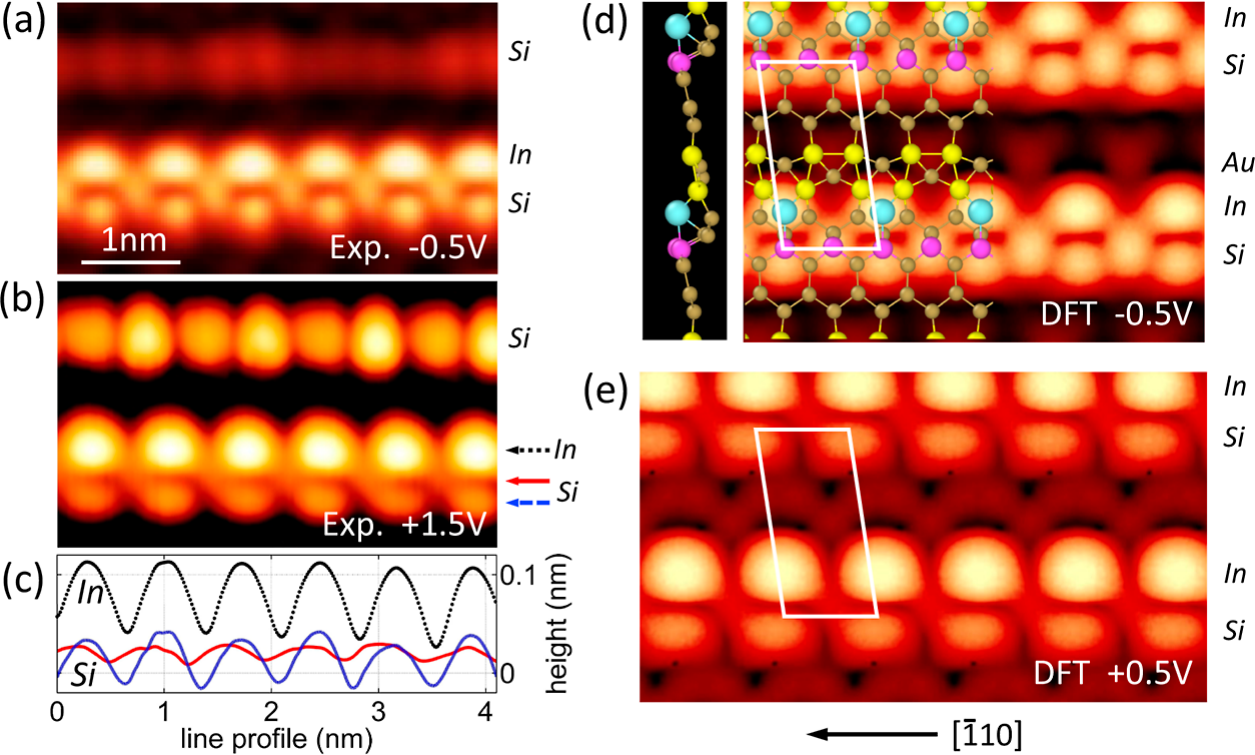
(a,b) STM topographic images measuring 4.6 × 2.8 nm of Si–In chains on the Si(553)–Au substrate, decorated with 0.05 ML In, captured at sample biases of −0.5 V (panel a) and +1.5 V (b). These images were recorded using a tunneling current of *I*_T_ = 20 pA. The positions of the In and Si chains in the system are indicated by arrows in (b), corresponding to the height profiles presented in (c). (d) and (e) display 4.6 × 1.8 nm DFT-simulated topographic images for sample biases of −0.5 and +0.5 V, respectively. (d) presents the structural model of the surface with In, Au, and Si atoms, alongside a side view of the atomic structure, in accordance with the DFT model described in ref (47). The white parallelograms in (d) and (e) mark the unit cell, and the  direction is indicated below (e).

First, the STM topography of the Si vicinal substrate decorated with In atoms is analyzed. Figure 1 displays atomically resolved experimental [panels (a) and (b)] and DFT-simulated images [panels (d) and (e)] of Si(553)–Au with In atoms, measured with both positive and negative sample biases indicated in each panel. The analysis is based on the lowest-energy structural model of In-decorated Si(553)–Au surface,^47^ where In atoms are bonded to Au dimers and also to every second step-edge Si atom. Panel (d) depicts the 2D calculated structural model and its side view along with the unit cell of the system. The experimental images (Figure 1a,b) display two distinct chains of bare step-edge Si atoms in the upper part of each panel and a Si–In chain in the lower section. Remarkably, the modulation observed in the bare Si chain under positive voltages conforms to the predicted periodicity of three lattice constants, as anticipated for the ground state within an infinitely long chain.^52^ Conversely, within the Si–In chain, both In and Si periodicities are equivalent to , where In atoms align opposite to Si atoms, forming a ladder-type structure for the Si–In chain.

The height profiles along the lines indicated by the arrows in panel (b) are analyzed in panel (c). The position of every second Si atom coincides with an In atom [black and blue curves in (c)]. However, the red profile line recorded between chains reveals additional Si atoms situated among the In sites. The maxima of the red curve in (c) do not precisely align between the In sites, indicating a small asymmetry in the position of these Si atoms. This asymmetry, discussed in ref (47), contributes to the zigzag-like structure of Si atoms and the SSH chain topography. Note that the electronic properties of the Si–In structure rely on the physical couplings between atomic states (not on the physical positions of the atoms). The STM topography images for positive- and negative-bias voltages reveal that each In atom forms bonds both with the opposite Si atom and simultaneously with the adjacent Si atom.

The simulated DFT topographic images in Figure 1d,e align well with the experimental ones. For positive biases, both In and Si chains exhibit  periodicity, determined by the occupation of every second unit cell along the terrace for In chains and caused by buckled dimers for Si chains. For negative biases, a zigzag geometry of Si orbitals with  periodicity is observed, where the pink dots representing Si atoms do not align precisely with the centers of bright circles underneath. Consequently, the Si–In chain yields a trimer unit cell composed of one In atom and two Si atoms. In the experimental image in Figure 1b, while the structure of buckled dimers is not resolved for a given positive STM voltage, the resulting double periodicity is distinctly visible.

DFT calculations, based on the lowest-energy structural model of In-decorated Si(553)–Au surface,^47^ validate the distance between In atoms along step edges at  = 0.768 nm, consistent with our experimental findings. The detailed analysis of the structural model indicates that In atoms form nearly equidistant (∼2.9 Å) bonds with Au dimers and neighboring or next-neighboring Si atoms, situated at a distance of 4.68 Å. However, the presence of In atoms disrupts the inversion symmetry along the step edges. This leads to variations in the In–Au bond lengths (2.87 vs 2.86 Å) and more significantly impacts the Si–Si distances within a chain (3.81 vs 3.90 Å). This discrepancy suggests distinct hopping integrals between neighboring Si atoms and nonzero nearest-neighbor and next-neighbor In–Si couplings.

**Figure 2:**
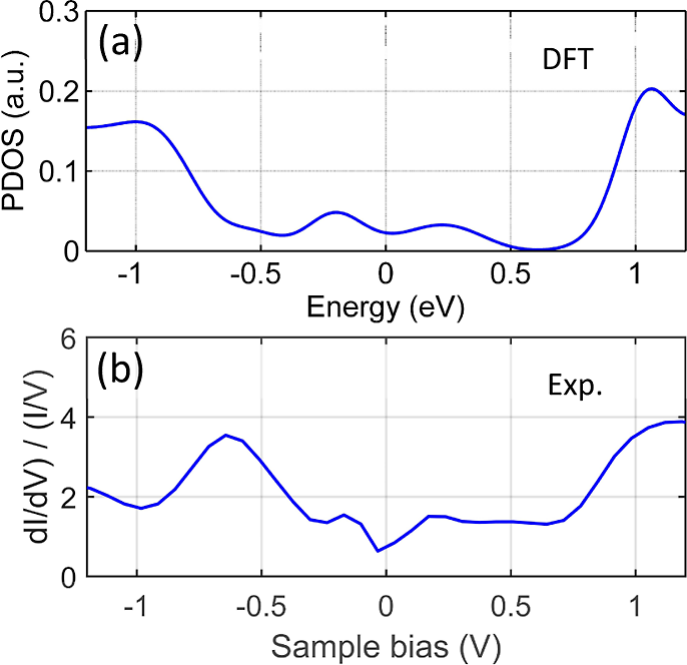
(a) Displays the calculated PDOS of two neighboring Si atoms within the Si chain’s unit cell, as depicted by the pink balls in Figure 1d, on the In-decorated Si(553)–Au substrate. The PDOS curve is an average across all local s, p_*x*,*y*,*z*_ orbitals at both Si sites. In (b), the experimental STS results show the normalized d*I*/d*V* derivative recorded over Si edge atoms situated in the middle of the Si chain, away from its ends. It is noteworthy that similar energy band features at both panels are observed above +1 V, near 0 V, and a band below −0.6 V.

The projected density of states (PDOS), averaged over all s and p orbitals, for two Si atoms in the primitive cell (two neighboring pink balls in the model, Figure 1d) is depicted in Figure 2a. These atoms, along with an In atom, constitute a unit cell of the system. The electronic structure of the Si atoms reveals energy states above +1.0 eV, states around +0.3 eV, and states lying below the Fermi level (below −0.6 eV). Notably, the PDOS related to each Si atom within the chain exhibits similar characteristics, indicating a strong hybridization within the system. These DFT calculations are compared with the normalized d*I*/d*V* derivative shown in Figure 2b. The curve was recorded over Si edge atoms positioned in the middle of a typical Si–In chain (away from the chain ends), reflecting the conditions under which the theoretical calculations were conducted (as DFT calculations cannot capture end-state physics and are performed for an infinite periodic system). Observing analogous features of the energy bands above +1 eV, near 0 eV, and below −0.6 eV, the agreement between the DFT calculations and the experimental spectroscopy results is evident, suggesting good agreement between the theory and experiment.

### Topological Nature of Si–In Atomic Chains

**Figure 3:**
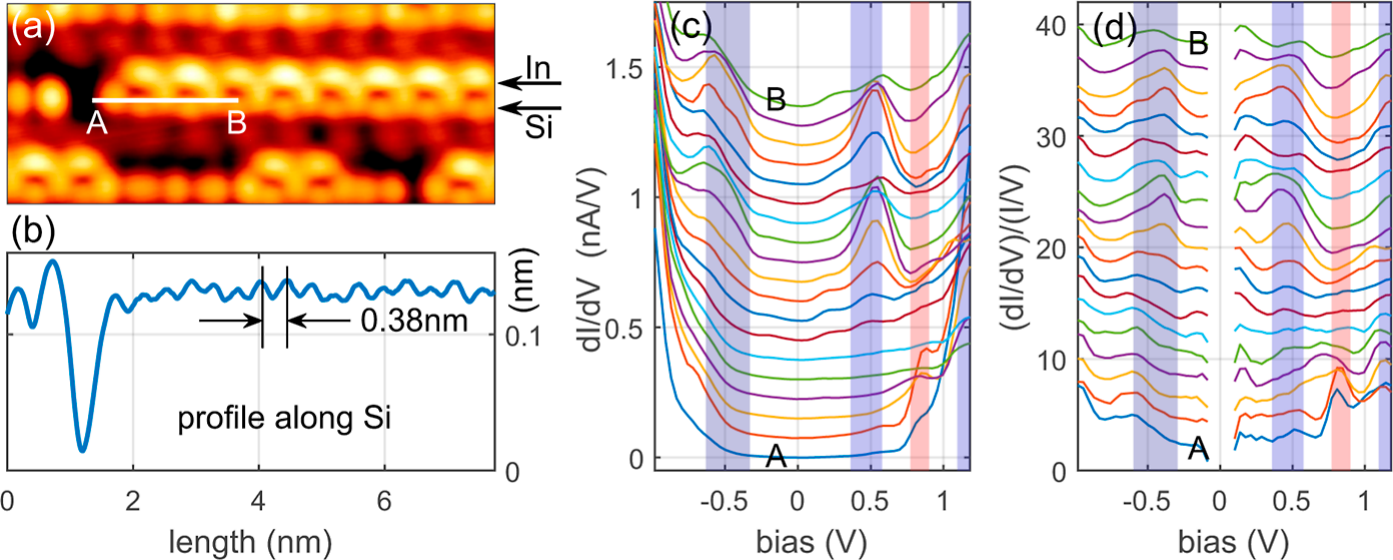
(a) High-resolution 7.8 × 3.4 nm topographic image of Si–In finite chain on Si(553)–Au surface recorded with the sample bias −1 V and a tunneling current of 200 pA. The arrows indicate positions of Si and In atoms in the Si–In chain. (b) Height profile along Si chain marked with the Si arrow in (a). The periodicity of this chain is the same as the periodicity of bulk Si  with an admixture of double periodicity, as clearly visible in (a). A deep minimum to the left of the profile line is attributed to Si atom vacancy. (c) d*I*/d*V* derivatives recorded over atoms marked by the white line in (a) from A point to B point at equidistant intervals. The curves are shifted vertically for clarity. (d) (d*I*/d*V*)/(*I*/*V*) spectra corresponding to d*I*/d*V* in (c). The vertical colored stripes in (c) and (d) represent characteristic energy bands (around +1.1, +0.5, and −0.5 eV) and topological state (about +0.75/+0.8 eV) of the structure under investigation.

To investigate the electronic properties of Si–In chains on the Si(553)–Au surface, STS measurements were performed, focusing on the chain end. Figure 3a displays a topographic image of the surface 7.8 × 3.4 nm recorded with a sample bias *U* = −1 V and tunneling current *I*_T_ = 200 pA. The Si atoms are observed as a chain exhibiting a  periodicity, with an atomic distance of 0.38 nm [refer to the profile line in Figure 3b recorded along the Si atoms, indicated by the black arrow in (a)]. Notably, the structure maintains its  periodicity with a unit cell comprising two Si sites and one In atom. While the unit cell appears topographically symmetrical along the chain, there is a phase difference in structures between adjacent terraces concerning the Si–In chain. This leads to minor asymmetry in couplings along the nearest Si atoms within the chain due to interactions with the substrate. Consequently, the Si chain presents distinct hybridization elements at every second site, representing the geometry of the SSH system. It is important to note that the deep minimum in the profile line in Figure 3b corresponds to the vacancy of the Si atom, enabling the analysis of end state effects in a finite chain, particularly focusing on the STS spectra.

It is well-known that the d*I*/d*V* derivative of a semiconductor sample or the (d*I*/d*V*)/(*I*/*V*) normalized derivative, is related, albeit not directly equivalent, to the local density of states (LDOS).^59,60^ Hence, in Figure 3, we have chosen to present two sets of derivatives: d*I*/d*V* in (c) and normalized d*I*/d*V* in (d). The tip placement during the acquisition of *I*(*V*) data is marked in Figure 3a by the white line A–B. The curves in panels (c) and (d) were recorded between points A and B at equidistant intervals. Consequently, the bottom curves correspond to the chain end, where the topological midgap state should be located. The d*I*/d*V* derivative and its normalized form, (d*I*/d*V*)/(*I*/*V*), displayed in Figure 3c,d, show electron states at +1.1 eV, approximately +0.45 eV, and around −0.5 eV, consistent with the electronic structure of Si–In chains discussed in Figure 2. These energy bands are marked in (c) and (d) with dark stripes. However, a compelling feature was observed at the end of the chain end. The curves near point A exhibit a gradually diminishing peak in d*I*/d*V* located at approximately +0.80 eV. This distinct peak is well-evident in the normalized d*I*/d*V* curves [panel (d)], particularly in the bottom curves, at an energy close to +0.75 eV. Interestingly, this state is absent in the STS data recorded toward the chain center, where the conductance spectra show local minima depicted by bright reddish stripes in (c) and (d). Notably, this state does not appear in other terrace sites neighboring the Si–In chain, indicating its specific association with the chain end, a characteristic feature attributed to the topological nature of the SSH chains.

### TB Calculations

**Figure 4:**
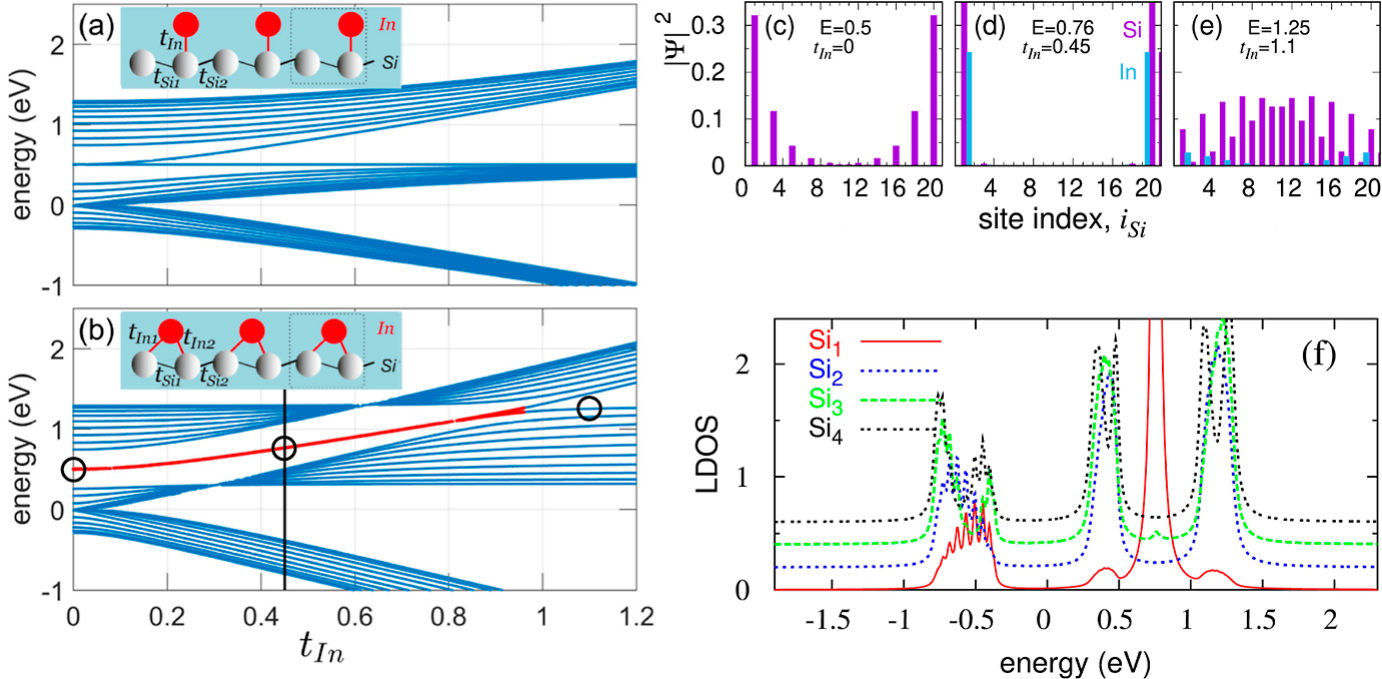
Energy spectra of the Si–In finite atomic chain are presented for the geometries depicted in the insets of (a) and (b), respectively, as a function of the Si–In coupling strength *t*_In_. In the schemes, In atoms are represented by red balls and Si atoms by gray balls. The system parameters are *t*_Si1_ = −0.5, *t*_Si2_ = −0.3, ε_Si_ = 0.5, ε_In_ = 0, *N*_Si_ = 20, *N*_In_ = 10, and Γ = 0.1. The vertical black line indicates the energy spectra structure for *t*_In_ = 0.45, utilized for LDOS calculations, while the red curve illustrates the modification of the topological state due to In atoms. (c) and (e) display the squares of the coefficients of the eigenfunctions for the bare Si chain (*t*_In_ = 0) in (c) and for the Si–In chain in the geometry presented in (b) for *t*_In_ = 0.45 (d) and *t*_In_ = 1.1 (e), corresponding to characteristic eigenenergies *E* = 0.5, 0.76, and 1.25 eV, respectively. The bar value corresponding to the end Si atoms on (d) reaches a value of 0.7 and extends beyond the vertical axis scale. These eigenenergies are marked by empty circles in (b). (f) exhibits the LDOS at the first four Si atoms in the chain from (b), Si_1–4_, as a function of energy, for *t*_In1_ = *t*_In2_ = 0.45. The curves in (f) are offset by 0.2, 0.4, or 0.6 from the bottom red curve for better visualization purposes.

To scrutinize the end state observed in the system under consideration, we conducted TB calculations, which effectively capture the fundamental physics of topological materials. The atomic schemes of the Si–In chains are illustrated in the insets of Figure 4a,b. The system comprises a Si atomic chain (gray balls) with side-attached In atoms (red balls) placed on the surface. Two potential configurations of In atoms along the Si chain are considered, both with low total energy obtained from DFT calculations. The model from panel (b) exhibits a local minimum energy slightly higher by only 110 meV (per unit cell) than the global energy minimum [observed for model (a)], rendering it realistic. For effective TB models, the physical positions of atoms are less critical; instead, the primary concern lies in the electronic interactions between atomic states. As evident from the STM topography images in Figure 1 for positive and negative biases, there exist bond couplings between each In atom and the opposite Si atom and asymmetric connections with two adjacent Si atoms, resulting in the effective models illustrated in Figure 4a,b. Consequently, in our model, In atoms can couple with either one Si atom or with two Si atoms, representing two extreme cases among possible intermediate situations where the In atom is asymmetrically coupled with two Si atoms. In these scenarios, the unit cell comprises three atoms, and the hopping integrals along the Si chain are denoted by *t*_Si1_ and *t*_Si2_; additionally, *t*_In1_ and *t*_In2_ describe the In–Si couplings within the system. This effective model represents an extended SSH chain with a trimer unit cell (SSH3) capable of revealing nontrivial topological states.

It is important to note that topological phases in 1D systems can exist in the presence of either chiral or inversion symmetry.^13,30,61^ The nature of the system under consideration holds the inversion symmetry (further described in Section Methods). Consequently, for a finite chain length, one anticipates the existence of protected topological states, potentially observable in the energy spectra or LDOS functions. Generally, for trimer lattices, the energy spectrum consists of three dispersive electron bands, with topological states potentially appearing between the bands within the energy gap regions.^19,24^ For a dimer coupled with one additional site, three separate states emerge, their positions depending on the dimer-adatom coupling strength, as observed in ref (40). A similar energy spectrum structure is expected for a trimer 1D lattice; except here, electron bands replace single states, with topological end states potentially manifesting within the energy gap. While in the original SSH chain, the topological modes precisely appear at the midgap energy, boundary topological states can also emerge at nonzero energies in extended SSH models.^14,18−24,62^ Moreover, it is worth noting that even in cases where the inversion symmetry is broken (due to disorders, defects, different on-site energies, asymmetrical couplings, or time-dependent perturbations), the localized nature of topological states can persist in such systems.^19,20,46^ Additionally, ladder-like atomic systems such as two coupled SSH chains^25−28^ and other SSH chain geometries^14,18−23,62^ also reveal nontrivial topological end states.

In Figure 4, we analyze the energy spectra of the Si–In chain for two configurations of In atoms [depicted in the insets in panels (a) and (b)] as a function of the In–Si coupling strength, *t*_In_. Both configurations depict two extreme models of the Si–In atomic arrangement. It is important to note that our focus here revolves around end-state effects, specifically considering a finite-length Si chain comprising *N*_Si_ = 20 Si atoms along with *N*_In_ = 10 In atoms. Here, we analyze the evolution of the Si chain band structure due to the coupling of In atoms.

For the Si chain decoupled from In atoms (*t*_In_ = 0), small differences exist in the *t*_Si1_ and *t*_Si2_ couplings (along the Si chain), resulting in a small energy gap and the presence of an end midgap state in the system. In the case of a one-to-one connection (Figure 4a) when In atoms are introduced (i.e., with increasing *t*_In_ coupling), this midgap state vanishes (merges into energy bands), and three electron bands emerge with two energy gaps between them. In this configuration, no electron states are present within the energy gaps, indicating that this system corresponds to the trivial SSH3 chain. However, the scenario changes in the second geometry, as depicted in Figure 4b. Noticeably, for a nonzero *t*_In_ coupling, three electron bands are observed within the system, and they progressively widen with the increase in the *t*_In_ parameter. Simultaneously, the SSH topological state emerges between the energy bands for smaller values of *t*_In_ (as seen in the red curve), signifying a more pronounced topological state within the system. Ultimately, as the coupling *t*_In_ increases further, the topological state merges into the band, leading the system to transition to a configuration characterized by trivial topology, with two energy gaps and the absence of midgap states. Comparing the outcomes displayed in Figure 4a,b, it is evident that model (a) lacks a boundary state in the presence of In atoms. In contrast, model (b) demonstrates an LDOS structure consistent with the experimental findings and DFT calculations, particularly for a nonzero coupling parameter *t*_In_. Moreover, this model accurately portrays the position of the topological boundary state. Given its close alignment with the experimental results, we select this model for further calculations.

To validate the topological characteristics of the midgap state in the Si–In chain, the eigenfunctions of the system were computed and are presented in Figure 4c–e. Panel (c) displays the eigenstate of the bare Si chain (*t*_In_ = 0) at an eigenenergy of *E* = 0.5 eV. This particular eigenstate exhibits the typical topological traits of the midgap state, akin to those seen in a regular SSH chain, where primarily the end atomic states contribute to the topological eigenfunction. The evolution of this topological state with an increase in the *t*_In_ coupling strength is evident in Figure 4d for *t*_In_ = 0.45 and *E* = 0.76 eV (the considered eigenenergies are marked by black circles in panel (b)). Here, the state involves solely the end Si and In atomic states, further confirming its topological nature. However, with a stronger *t*_In_ coupling, as shown in panel (e) for *t*_In_ = 1.1 and *E* = 1.25 eV, the eigenstate near the energy band comprises all atomic states of the chain, indicating the loss of its nontrivial topological properties.

The presence of a topological state within the system should also be manifested in the LDOS characteristics. Thus, it is crucial to analyze the LDOS function at various chain sites. These features are directly associated with the STM spectroscopy results (illustrated in Figure 3). In Figure 4f, we display the LDOS values at the first four Si atoms in the chain, representing the end sites. The LDOS structure for all Si atoms exhibits three electron bands around *E* = +1.1 + 0.4 eV and below −0.5 eV, creating two energy gaps between these bands. However, at the first Si atom within the chain, an additional electron state emerges within the energy gap, approximately at *E* = +0.75 eV (this state appears also at the last chain site as we consider here a symmetrical system with an even number of Si atoms). Notably, such an LDOS peak is absent in the interior Si atoms, consistent with both our DFT calculations (as depicted in Figure 2) and the STM spectroscopy results (as seen in Figures 2 and 3). This observation suggests that this particular topological state is not present within the chain’s interior atoms but exclusively manifests at the end Si atoms. Consequently, the analysis of LDOS, along with the energy spectra results and eigenfunction distributions, collectively indicates that the trimer Si–In chain exhibits nontrivial SSH topological properties.

### Odd Number of Si Atoms in Atomic Chain

**Figure 5:**
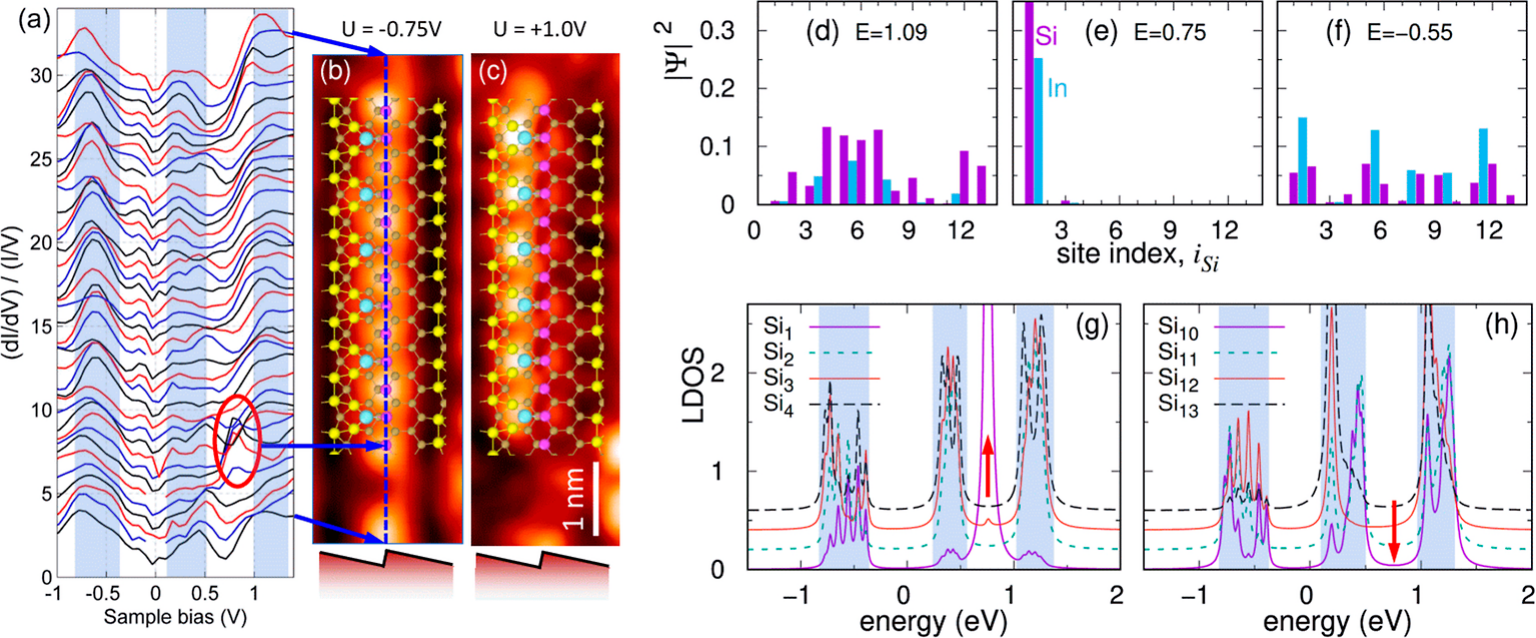
(a) Normalized (d*I*/d*V*) derivatives obtained from measurements along the finite odd-length Si–In chain, indicated by the dashed blue line in the STM topographic image shown in (b). The arrows highlight specific positions where STS spectra were recorded, with other curves taken at equidistant intervals between these points. The curves are vertically shifted for clarity. Colored vertical stripes in (a) represent characteristic energy bands of the system (around +1.1, +0.5, and −0.5 eV), while the red oval highlights the topological state within the conductance curves. (b) and (c) present topographic images featuring the atomic structure of the Si–In finite chain comprising *N*_Si_ = 13 Si atoms and *N*_In_ = 6 In atoms, recorded at sample biases of −0.75 (b) and +1.0 V (c), with a tunneling current of 200 pA. (d–f) display TB calculations of the Si–In chain eigenfunctions for eigenenergies *E* = 1.09 eV (d), *E* = 0.75 eV (e), and *E* = −0.55 eV (f) using the same parameters as in Figure 4b and for *N*_Si_ = 13, *N*_In_ = 6, and *t*_In_ = 0.45. The bar value corresponding to the first Si atom on (e) reaches a value of 0.74 and extends beyond the vertical axis scale. LDOS at both chain ends are depicted in (g) for the first four Si atoms and in (h) for the last four Si sites. The curves in (g) and (h) are shifted from the bottom curve for better visualization.

To establish compelling evidence regarding the topological nature of self-assembled Si–In chains on the Si(553)–Au surface, we conducted additional experimental and theoretical investigations. A comprehensive analysis was undertaken on the chain structured with an odd number of Si atoms in the SSH geometry. In such a chain, the anticipation is that topological states would manifest only at one end of the chain and remain absent on the opposite side due to the absence of one Si atom at the end cell. It is an inherent property that normal boundary states always appear at both chain ends, irrespective of the chain’s parity, which serves to distinguish between the topological and trivial phases of the system. In Figure 5a, we present the STS data of normalized (d*I*/d*V*) derivatives for a finite chain composed of *N*_Si_ = 13 Si atoms and *N*_In_ = 6 In atoms. The topographic images of this chain, along with the structural atomic model, are depicted in panel (b) for the bias of −0.75 V and in panel (c) for +1.0 V. The normalized conductance spectra shown in panel (a) were recorded along the line of Si atoms marked by the dashed blue line in (b) (measured at equidistant points between the arrows). Three energy bands of the system (around +1.1 +0.5, and approximately −0.5 eV) marked by dark vertical stripes are discernible, consistent with our previous observations. However, select curves exhibit additional d*I*/d*V* end peaks at a sample voltage of approximately +0.75 V (highlighted with a red oval in Figure 5a), notably associated only with one end of the chain. Importantly, such peaks were not observed at the opposite side of the odd-length Si–In chain.

To elucidate our experimental findings, we conducted TB calculations of the system’s wave functions (Figure 5d–f) and LDOS at both chain ends [panels (g) and (h)] for the SSH-like Si–In chain. The LDOS structure for all Si atoms reveals three energy bands colored vertical stripes corresponding to the same energies observed in the experimental conductance curves presented in panel (a). However, only one site in the chain (Si_1_ end atom) exhibits the manifestation of a topological state at an energy of +0.75 eV [panel (g)], while this peak is not observed at the other chain end for the last site (Si_13_), as is indicated by the red arrows. The confirmation of the system’s topological nature is further supported by calculations of the eigenfunctions analyzed in panels (d–f) for the eigenenergies *E* = 1.09 eV (d), *E* = 0.75 eV (e), and *E* = −0.55 eV (f). The eigenstates localized within the energy bands [panels (d) and (f)] encompass all atomic Si and In states. However, the eigenstate within the energy gap [panel (e)] solely comprises atomic states localized at one chain end, confirming its topological nature. The experimental and theoretical results presented in Figure 5 strongly indicate an additional topological state at only one chain end around the energy *E* = +0.75 eV. This signifies the topological character of this state, reinforcing our argument regarding the topological phase of the self-assembled Si–In system.

## Summary and Conclusions

In this study, we propose a self-assembly technique for creating an SSH nontrivial topological system using real Si and In atoms. Our approach involves adding In atoms to a Si chain, resulting in a robust system featuring energy gaps with topologically nontrivial states observed at the chain ends. We investigated the electronic properties of Si–In chains formed on a Si(553)–Au surface using the STM method, complemented by DFT and TB calculations. The self-assembled chains were fabricated by decorating Si(553)–Au with In atoms, where the unit cell of these chains comprises two Si atoms and one side-attached In atom. A primary outcome of this study is the identification of such a trimer chain as an atomic realization of the extended SSH model with topological end states. STM spectroscopy results revealed that each chain is characterized by three electron bands and two energy gaps. Crucially, quantum states exclusively emerge at the chain ends within the energy gap region—a distinctive feature of nontrivial topological modes. DFT calculations were conducted to confirm the positions of In and Si atoms within the chain on the Si(553)–Au substrate, analyze the spatial arrangement of Si orbitals, and study the PDOS for the chain. To explain the appearance of topological states in the system, we proposed a TB model for the trimer SSH chain, analyzing the energy spectra together with LDOS functions and eigenfunction distributions. Our theoretical findings exhibited satisfactory qualitative agreement with the experimental observations.

We analyzed an odd-length Si–In chain, where topological states are expected to appear only at one chain end, in contrast to ordinary end states existing at both ends of the system. Our experimental and theoretical results confirmed this prediction, indicating the topological nature of the end state and reinforcing our argument regarding the topological phase of the self-assembled Si–In system. It is worth noting that the Si–In topological system analyzed in this study is fabricated not from atomic vacancies but from real atoms through a self-organizing method. Therefore, our findings hold practical significance, particularly considering the stability of such chains. Additionally, the fabrication of such topological systems can be entirely controlled by the amount of evaporating atoms, strongly determining the chain lengths and the number of topological chains on the substrate.

## Methods

### Experimental Setup

The experiments were carried out in the ultrahigh vacuum system with a base pressure in the middle of the range of 10^–11^ mbar. The system was equipped with a reflection high-electron energy diffraction (RHEED) diffractometer, OMICRON LT STM/AFM apparatus, Au and In deposition sources, and a precise quartz microbalance sensor. N-type Si(553) samples with a specific resistivity of 0.002 ÷ 0.01 Ω·cm were cleaned according to the standard procedure for Si samples. After several hours of degassing, the samples were finally cleaned at a temperature of about 1500 K using a DC flash. Au atomic chains were prepared by depositing 0.44 ML (monolayer) of Au on a sample at room temperature (RT), followed by a short anneal at about 1100 K followed by gradual reduction to RT over 2 min. In this article, one monolayer denotes the density of Si(111) surface atoms (7.84 × 10^14^ atoms/cm^2^). Next In was deposited with doses ranging from 0.03 to 0.1 ML on the sample kept at RT. Scanning tunneling topography and STS measurements were carried out at 77.4 K. The d*I*/d*V* plots were calculated numerically from recorded *I*(*V*) curves. All sample preparation steps were controlled with the RHEED diffractometer.

### DFT Method

The DFT calculations have been performed using the VASP^63,64^ and the PBEsol correlation-exchange functional.^65^ In electronic structure calculations, the HSEsol hybrid functional has been utilized.^66^ A kinetic energy cutoff of 340 eV is for the plane wave expansion of single-particle wave functions. The Brillouin zone was sampled by 8 × 4 × 1 Monkhorst–Pack *k*-points grid.^67^ The convergence for the total energy was chosen as 10^–6^ eV between subsequent iteration steps, and the maximum force allowed on each atom during the geometry optimization was less than 0.01 eV/Å. These parameters were tested and optimized to obtain well-converged total energies of the system. The Si(553)–Au system has been built according to the structural model of ref (47) and consists of four Si double layers, H-passivated at the bottom. A vacuum space of 19 Å was introduced to prevent any unphysical interaction between the periodic images of the slab. A supercell with a 2 × 1 periodicity was considered, in agreement with the experimental conditions.

### TB Model

The model chain comprises *N*_Si_ Si sites with site-attached *N*_In_ In atoms and can be described by the following Hamiltonian written in terms of a second quantization notation
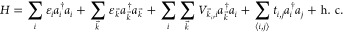
1The summation over *i* runs across all Si and In atoms, while ⟨*i*, *j*⟩ represents the summation over neighboring sites. Here, *a*_*i*_^†^,*a*_*i*_, ,  denote the creation/annihilation operators at the *i*th site (Si or In) of the chain or the surface electrode in the  state, respectively. The on-site energy level for Si atoms is denoted by ε_*i*_ = ε_Si_, and for In atoms, it is ε_*i*_ = ε_In_.  corresponds to possible electron energies in the surface. The parameter  represents the hybridization element between the surface and chain states, while *t*_*i*,*j*_ is responsible for the couplings between the nearest neighbor chain sites: for Si atoms, *t*_*i*,*j*_ = *t*_Si1_ within the primitive cell, and *t*_*i*,*j*_ = *t*_Si2_ between Si atoms from the neighboring cells. Each In atom is coupled to only one or two Si sites, which is noted as *t*_*i*,*j*_ = *t*_In1/In2_.

Electronic properties of the system are analyzed within the framework of Green’s function method. The LDOS for each site is obtained using the relation , where *G*_*ii*_^r^(*E*) represents the retarded Green function associated with the *i*th site of the chain and can be computed using the equation of motion technique.^46,68^ This yields algebraic complex equations for *G*^r^, expressed as , where *Î* represents the unit matrix. The elements of the *Â* matrix are defined as . The chain-surface coupling, , generally relies on the arrangement of surface atoms and electron localization/delocalization in the substrate. In our considered system, resembling a semiconductor-like surface, we approximate the chain-surface coupling within a wide band approximation as energy-independent, such that Γ_*ij*_(*E*) = Γδ_*ij*_. It is assumed here that electron–electron interactions do not significantly impact the system and can be captured by an effective shift of the chain on-site energies without leading to correlation effects, which is justifiable for the considered system. Consequently, both spin directions are treated independently of each other, and therefore, the spin indices are not explicitly expressed.

In our TB calculations, all energies are expressed in units of Γ^0^ = 1. Hence, for Γ^0^ = 1 eV, the coupling strength between atomic sites remains below 1 eV, while the chain-surface coupling is set at Γ = 0.1 eV.

The parameters within the TB model were fine-tuned to align with the hopping integrals observed in real Si–In chains. These parameters are derived from the couplings identified through the energy dispersion relation obtained from our experimental observations and DFT calculations. All parameters within this model should be regarded as effective parameters tailored to best replicate the experimental and DFT outcomes. It is important to note that minor alterations in the system parameters yield comparable results and do not lead to divergent conclusions. The reference energy point is the Fermi energy of the surface electrode, set as *E*_F_ = 0 eV, and the case examined assumes zero temperature, *T* = 0 K. For the system under consideration and assuming periodic boundary conditions along the chain decoupled from the substrate, given the discrete translational invariance, one can conduct a Fourier transform of the annihilation/creation operators , where ϕ_*n*_=(*a*_Si1,*n*_,*a*_Si2,*n*_,*a*_In,*n*_)^*T*^ and the parameter *n* represents the unit cells. In the reciprocal space, the Hamiltonian can be expressed as follows: *H* = ∑_*k*0_ϕ_*k*0_^†^*H*(*k*_0_)ϕ_*k*0_, where the matrix Hamiltonian for ε_*i*_ = 0 takes the following form
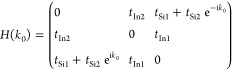
The given Hamiltonian adheres to inversion symmetry, as , where  represents a 3 × 3 matrix akin to the role of the Pauli matrix σ_*x*_, satisfying  and . Hence, such a system allows for the existence of protected topological states. Please note that the system under consideration, for *t*_In_ = 0, represents the SSH model in either the trivial or nontrivial topological phase (depending on the *t*_Si1_ and *t*_Si1_ parameters). For such a system, well-known topological invariants exist.^13^ For instance, the winding number assumes values of either 1 or 0. With the presence of In atoms forming a unit cell consisting of three atoms, although the Hamiltonian maintains time reversal symmetry, chiral symmetry is not preserved in this scenario. Consequently, the winding number and Zak phase are not considered reliable topological numbers in this context.^19,24^ Nevertheless, we can analyze the evolution of the SSH topological state in the presence of In atoms.
